# Vulvar findings and sexual dysfunction in women with systemic sclerosis

**DOI:** 10.1016/j.jdin.2026.05.019

**Published:** 2026-05-30

**Authors:** Marine Mercipinetti, Ségolène Delmas-Lanta, Guillaume Chaby, Vincent Goeb, Jean Schmidt, Aloïs Reilhac, Jean-Philippe Arnault

**Affiliations:** a Department of Dermatology, Amiens-Picardie University Medical Center, Amiens, France; b Department of Gynecology, Amiens-Picardie University Medical Center, Amiens, France; c Department of Rheumatology, Amiens-Picardie University Medical Center, Amiens, France; d Department of Internal Medicine, Amiens-Picardie University Medical Center, Amiens, France; e Nouvelle-Aquitaine Regional Health Agency (ARS), Bordeaux, France

**Keywords:** lichen sclerosus, sexual dysfunction, squamous cell carcinoma, systemic sclerosis, vulvar examination, vulvar pathology, vulvodynia

*To the Editor:* Mucosal involvement in systemic sclerosis (SSc) is well recognized; however, vulvar manifestations and associated sexual dysfunction remain poorly described and likely underdiagnosed.^1^^,^^2^ We aimed to characterize vulvar involvement in SSc and evaluate its impact on sexual quality of life. We conducted a cross-sectional, single-center study included women aged ≥18 years old with SSc fulfilling the 2013 American College of Rheumatology/European League Against Rheumatism classification criteria, who were evaluated between 2013 and 2023.^3^ Patients were referred for a standardized consultation conducted by the same dermatologist and gynecologist, both specialized in vulvar disorders. The evaluation included a structured interview, a physical examination, and completion of the Female Sexual Function Index questionnaire. Sexual dysfunction defined as Female Sexual Function Index score ≤26.55.^4^

Of the 111 eligible patients, 41 were included in the analysis. The median [IQR] age was 64 [39-82] and the median disease duration was 12 years [4-33]. Most patients had limited cutaneous SSc (83%) and a low Rodnan score (median [IQR] 6 [2-9]); 36 (88%) were postmenopausal.

The most common finding was vulvar synechiae (adhesion or fusion of the labia minora and clitoral hood), observed in 20 (49%) patients (Fig 1). In contrast, vulvar cutaneous sclerosis was less frequent (17%). Patients with vulvar synechiae were significantly older (*P* = .011) and more frequently postmenopausal (*P* = .048). Importantly, the presence of synechiae was not associated with markers of SSc severity or disease duration, including the modified Rodnan skin score (*P* = .5) and disease duration (*P* = .8) (Table I). Notably, none of these patients were aware of these abnormalities, which were often asymptomatic. Vulvodynia was present in 12 (29%) patients, and 7 of the latter had concomitant synechiae. Sexual dysfunction was highly prevalent, affected 25 of 32 (78%) patients who respond.

**Fig 1:**
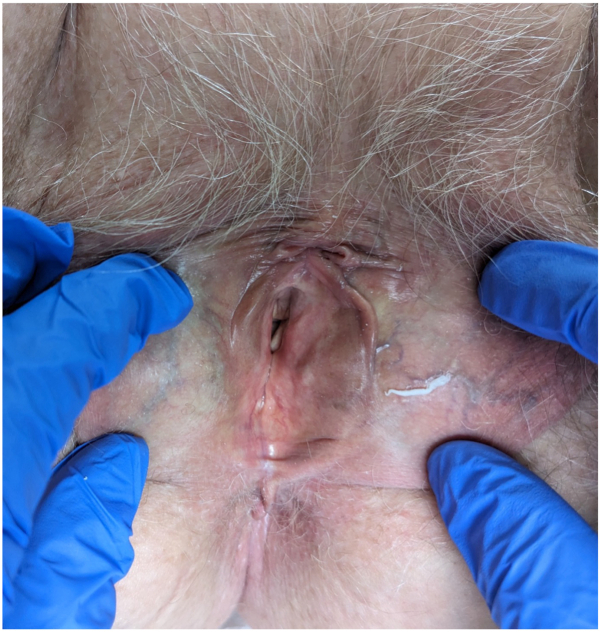
Bilateral synechiae of the labia minora, in the absence of obvious sclerosis associated with moderate narrowing of the introitus in a postmenopausal woman not engaging in penetrative sexual activity.

**Table I:** Demographic and clinical characteristics of patients with systemic sclerosis, stratified by the presence of vulvar synechiae

	Total, *N* = 41∗	95% CI	No vulvar synechiae, *N* = 21	95% CI	Vulvar synechiae, *N* = 20	95% CI	*P* value
Age, median [IQR]	64 (55, 74)		58 (51, 68)	[54, 65]	68 (63, 75)	[65, 72]	.011∗
Smoking status, *n* (%)							.6†
No	37 (90)	[76, 97]	18 (86)	[63, 96]	19 (95)	[73, 100]	
Yes	4 (9.8)	[3,2, 24]	3 (14)	[3,8, 37]	1 (5)	[0,26, 27]	
Duration of systemic sclerosis progression, median [IQR]	12 (8, 17)		12 (10, 16)	[11, 16]	12 (6, 18)	[9.6, 17]	.8∗
Rodnan score, median [IQR]	6 (2, 9)		6 (4,11)	[4,7, 11]	6 (2, 7,2)	[3.8, 8]	.5∗
Menopause, *n* (%)							.048†
No	5 (12)	[4,6, 27]	5 (24)	[9,1, 48]	0 (0)	[0, 20]	
Yes	36 (88)	[73, 95]	16 (76)	[52, 91]	20 (100)	[80, 100]	
Systemic sclerosis type, *n* (%)							.4†
Diffuse	34 (83)		19 (90)	[71.1-97.3]	15 (75)	[53.1-88.8]	
Limited	3 (7.3)		1 (4.8)	[0.8-22.7]	2 (10)	[2.8-30.1]	
*Sine scleroderma*	4 (9.8)		1 (4.8)	[0.8-22.7]	3 (15)	[5.2-36.0]	

*CI*, Confidence interval; *IR*, interquartile range.

∗ According to Wilcoxon’s test.

† According to Fisher’s exact test.

These findings support consideration of genital examination in women with SSc, particularly when vulvar symptoms or sexual dysfunction are present. The study had several limitations. While the classic clinical other hallmarks of LS (erythema, erosions, subepithelial hemorrhages, and shiny white atrophic plaques) were not observed, but lichen sclerosus cannot be excluded definitively without histopathological confirmation. Secondly, women with vulvar synechiae were older and more likely to be menopaused that women without vulvar synechiae. These factors can contribute to moderate anatomical changes in vulvar structures. Without an age-matched postmenopausal control group without systemic sclerosis, the study cannot really determine whether synechiae are attributable to systemic sclerosis, postmenopausal vulvovaginal change, sexual inactivity, and occult lichen sclerosus. Thirdly, many patients, particularly younger ones, failed to attend a consultation because they were reluctant to undergo a further gynecological examination. Failure to attend and fear of pelvic examinations are universal issues in younger women.^5^ This sampling bias is also due to the refusal of patients for lack of symptoms. Lastly, the study’s cross-sectional design prevented us from assessing changes over time.

In conclusion, vulvar involvement appears to be common but underrecognized in women with SSc: a genital examination and an assessment of sexual function in this population could be of interest.

### Declaration of generative AI and AI-assisted technologies in the writing process

None.

## Conflicts of interest

None disclosed.
